# P-2048. Evaluating the Impact of Removing Urine Culture Testing from Emergency Department Order Sets

**DOI:** 10.1093/ofid/ofaf695.2212

**Published:** 2026-01-11

**Authors:** Tsiani R Adopleh, Daawar Chaudhry, Roseline Idoko, Maricelle Monteagudo-Chu, Adam Wos, Lindsay Lindsay, Alan Kaell, David Galinkin, Anas Sawas

**Affiliations:** Zucker School of Medicine, Northwell Health at Mather Hospital, Port Jefferson, NY, USA, Port Jefferson, NY; Zucker School of Medicine, Northwell Health at Mather Hospital, Port Jefferson, NY, USA, Port Jefferson, NY; Zucker School of Medicine, Northwell Health at Mather Hospital, Port Jefferson, NY, USA, Port Jefferson, NY; Mather Hospital, Port Jefferson, New York; Northwell Health Mather Hospital, Port Jeffereson, New York; Mather Hospital, Port Jefferson, New York; Northwell Hofstra ZSOM, Port Jefferson, New York; Mather Hospital, Port Jefferson, New York; Mather Hospital, Port Jefferson, New York

## Abstract

**Background:**

Unnecessary urine cultures, especially in asymptomatic patients, contribute to asymptomatic bacteriuria overtreatment, inappropriate antibiotic use, increased adverse effects, and delayed discharges. Our quality improvement (QI) initiative addressed electronic medical record (EMR)-driven urine culture overordering in the ED stemming from defaulted pre-checks in syndromic order sets (OS).

The aim of the QI project is to reduce unnecessary urine culture orders by at least 10% within 1 month after removing the default urine culture orders and educating the ED staff on the appropriate indications for urine culture ordering.

**Table 1.**
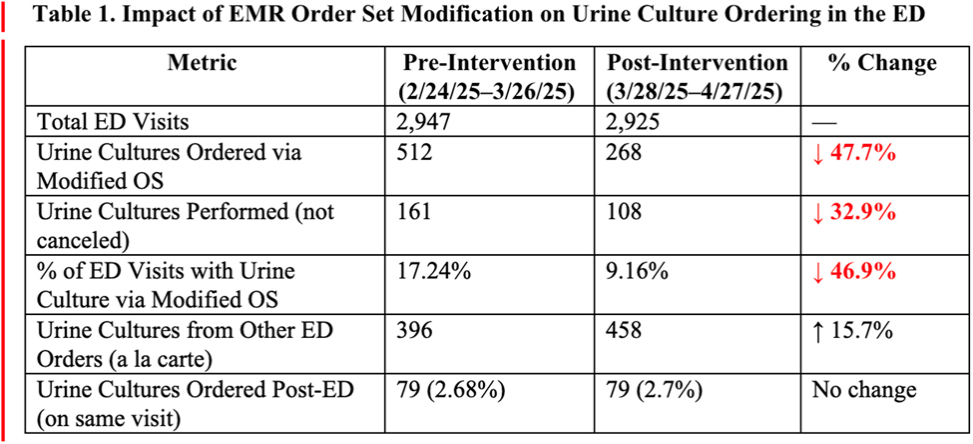
Impact of EMR Order Set Modification on Urine Culture Ordering in the ED

**Table 2.**
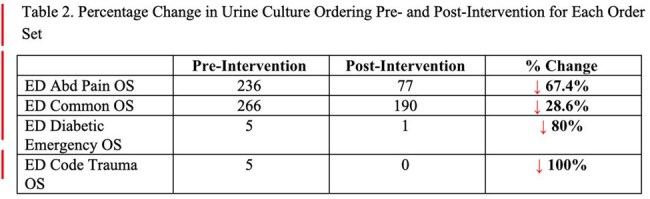
Percentage Change in Urine Culture Ordering Pre- and Post-Intervention for Each Order Set

**Methods:**

We modified several OS by unchecking pre-selected urine cultures in the ED Abdominal Pain, ED Bariatric, ED Code Trauma, and ED Diabetic Emergency OS. Urinalysis (UA) and urine culture orders were decoupled in the Common OS. ED staff received education on appropriate urine culture ordering. We compared urine culture ordered and performed using these modified OS one month pre- and post-intervention. Urine cultures ordered outside these OS were also evaluated.

**Results:**

In the pre-intervention period, 869 of 2,947 ED visits resulted in at least one urine culture order, with 512 (17.24%) originating from the targeted OS. In the post-intervention period, 720 of 2,925 ED visits included urine cultures, with 268 (9.16%) ordered from the targeted OS. There was a 48% decrease in urine cultures ordered and a 33% decrease in urine cultures performed from the targeted ED OS post-intervention (see Table 1). The breakdown of the urine culture ordered before and after intervention from the OS are summarized in Table 2. There was no urine culture ordered from the ED Bariatric OS in the pre- and post-intervention period.

**Conclusion:**

Our reduced unnecessary urine culture orders by over 45% in a short timeframe. The increase in urine cultures ordered outside the OS during the post-intervention phase may be attributed to providers making conscious decisions in determining when a urine culture is necessary. EMR order set optimization and clinical decision support are powerful tools for enhancing diagnostic stewardship, reducing antibiotic overuse, and improving care efficiency. Sustained implementation and provider education are essential to maintain progress.

**Disclosures:**

All Authors: No reported disclosures

